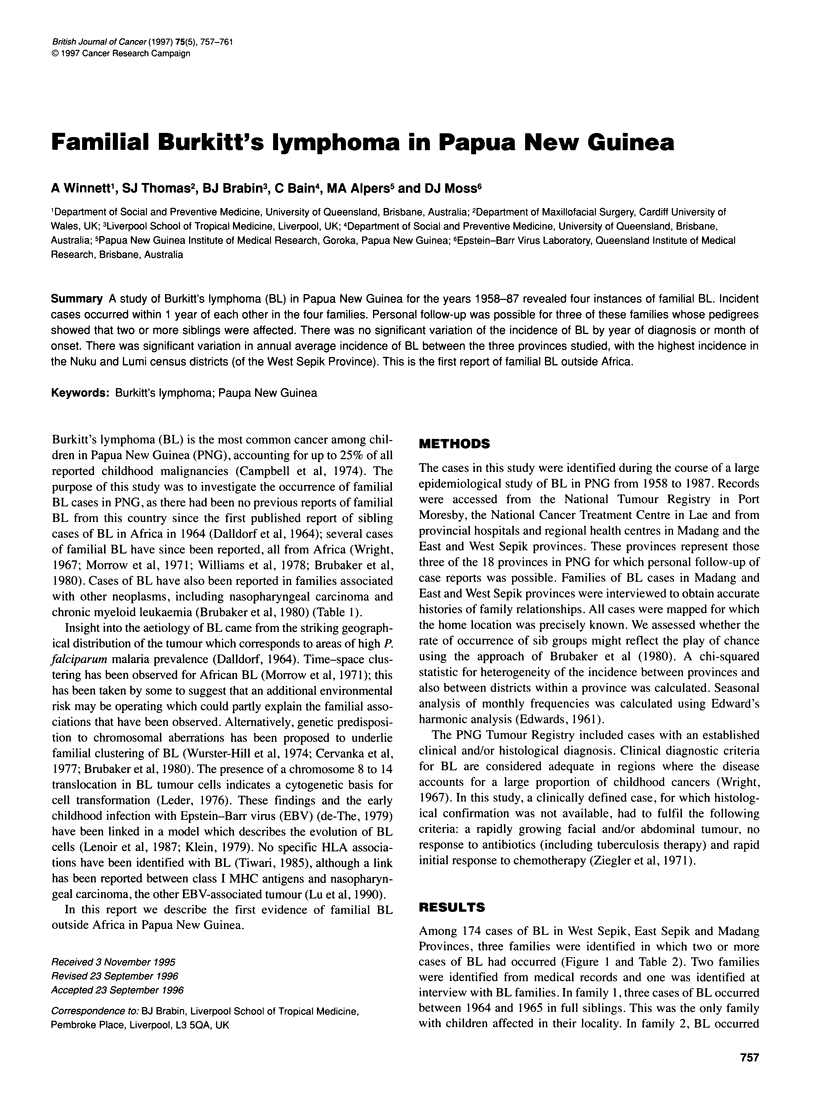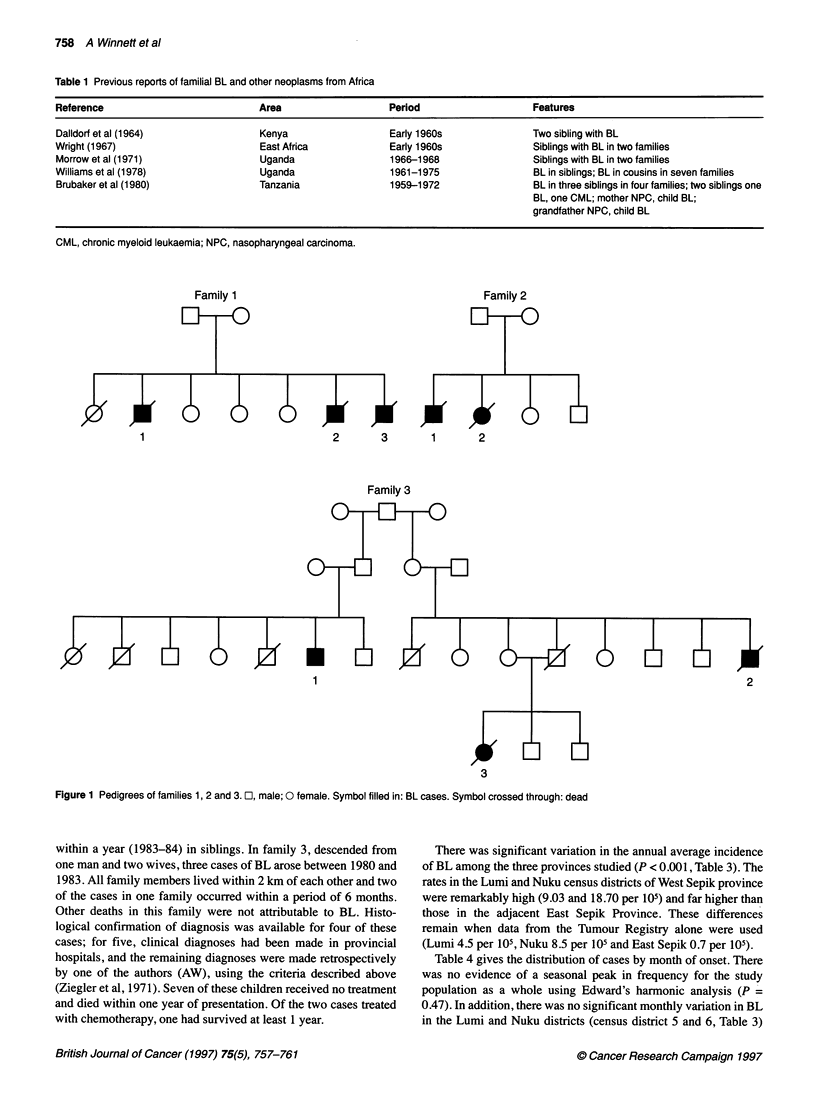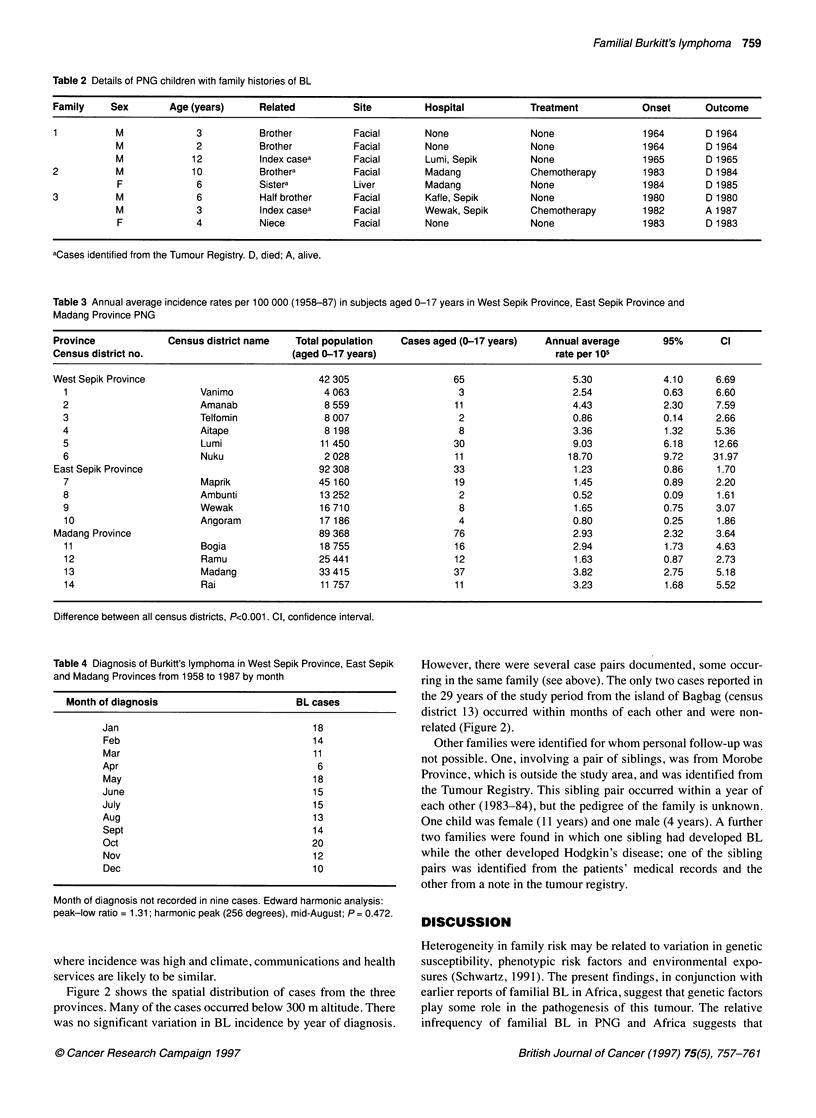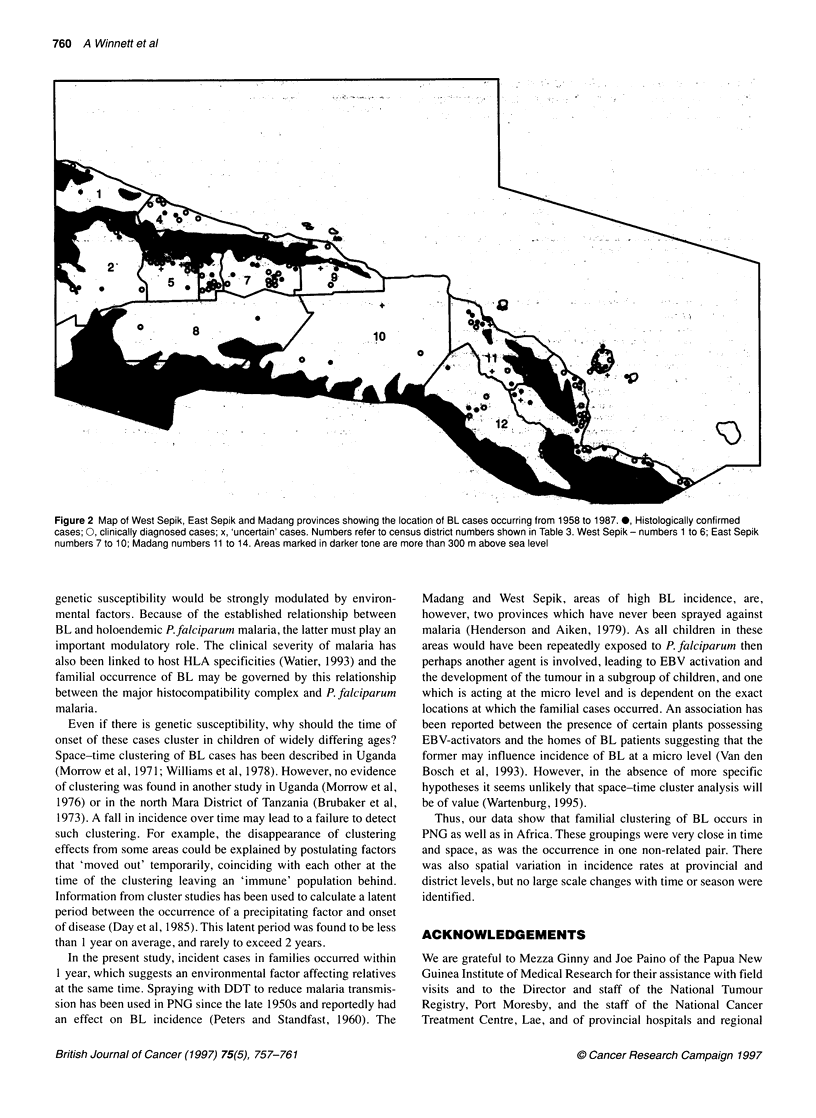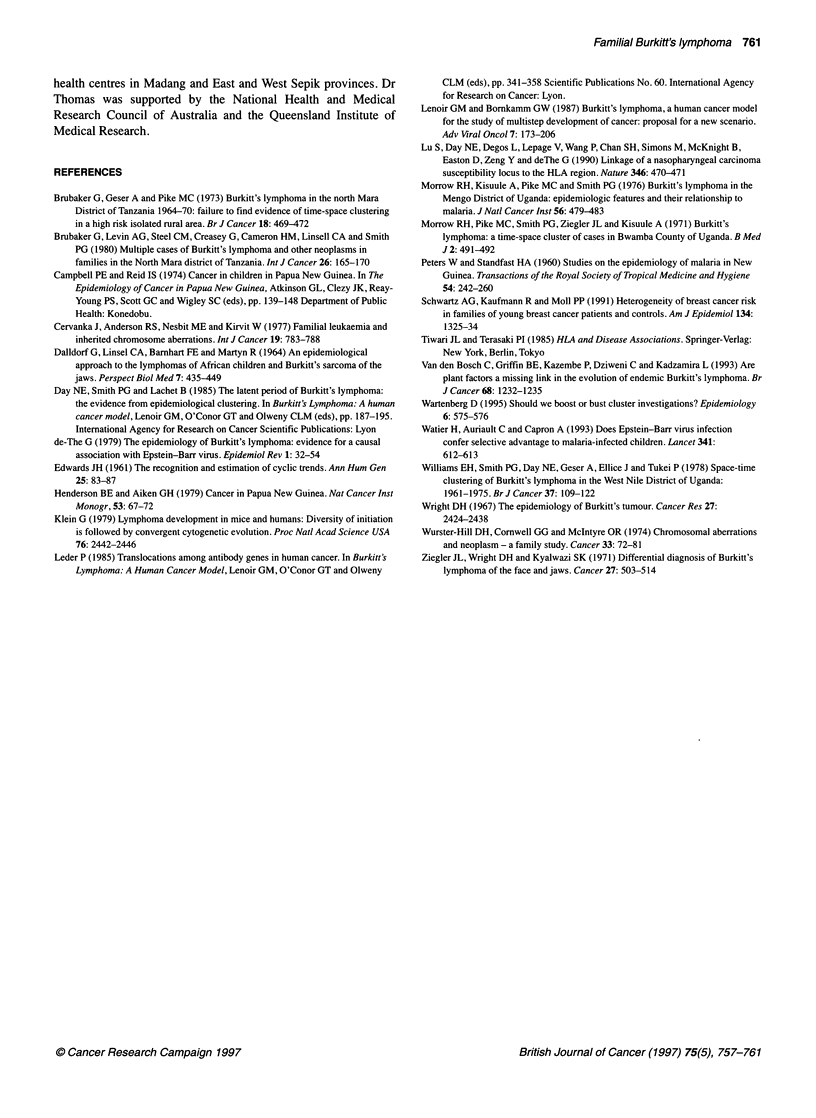# Familial Burkitt's lymphoma in Papua New Guinea.

**DOI:** 10.1038/bjc.1997.134

**Published:** 1997

**Authors:** A. Winnett, S. J. Thomas, B. J. Brabin, C. Bain, M. A. Alpers, D. J. Moss

**Affiliations:** Department of Social and Preventive Medicine, University of Queensland, Brisbane, Australia.

## Abstract

A study of Burkitt's lymphoma (BL) in Papua New Guinea for the years 1958-87 revealed four instances of familial BL. Incident cases occurred within 1 year of each other in the four families. Personal follow-up was possible for three of these families whose pedigrees showed that two or more siblings were affected. There was no significant variation of the incidence of BL by year of diagnosis or month of onset. There was significant variation in annual average incidence of BL between the three provinces studied, with the highest incidence in the Nuku and Lumi census districts (of the West Sepik Province). This is the first report of familial BL outside Africa.


British Journal of Cancer (1997) 75(5), 757–761
© 1997 Cancer Research Campaign

### Familial Burkitt's lymphoma in Papua New Guinea

**A Winnett[1], SJ Thomas[2], BJ Brabin[3], C Bain[4], MA Alpers[5] and DJ Moss[6]**

[1]Department of Social and Preventive Medicine, University of Queensland, Brisbane, Australia; [2]Department of Maxillofacial Surgery, Cardiff University of Wales, UK; [3]Liverpool School of Tropical Medicine, Liverpool, UK; [4]Department of Social and Preventive Medicine, University of Queensland, Brisbane, Australia; [5]Papua New Guinea Institute of Medical Research, Goroka, Papua New Guinea; [6]Epstein–Barr Virus Laboratory, Queensland Institute of Medical Research, Brisbane, Australia

**Summary** A study of Burkitt's lymphoma (BL) in Papua New Guinea for the years 1958–87 revealed four instances of familial BL. Incident cases occurred within 1 year of each other in the four families. Personal follow-up was possible for three of these families whose pedigrees showed that two or more siblings were affected. There was no significant variation of the incidence of BL by year of diagnosis or month of onset. There was significant variation in annual average incidence of BL between the three provinces studied, with the highest incidence in the Nuku and Lumi census districts (of the West Sepik Province). This is the first report of familial BL outside Africa.

**Keywords:** Burkitt's lymphoma; Paupa New Guinea

Burkitt's lymphoma (BL) is the most common cancer among children in Papua New Guinea (PNG), accounting for up to 25% of all reported childhood malignancies (Campbell et al, 1974). The purpose of this study was to investigate the occurrence of familial BL cases in PNG, as there had been no previous reports of familial BL from this country since the first published report of sibling cases of BL in Africa in 1964 (Dalldorf et al, 1964); several cases of familial BL have since been reported, all from Africa (Wright, 1967; Morrow et al, 1971; Williams et al, 1978; Brubaker et al, 1980). Cases of BL have also been reported in families associated with other neoplasms, including nasopharyngeal carcinoma and chronic myeloid leukaemia (Brubaker et al, 1980) (Table 1).

Insight into the aetiology of BL came from the striking geographical distribution of the tumour which corresponds to areas of high *P. falciparum* malaria prevalence (Dalldorf, 1964). Time–space clustering has been observed for African BL (Morrow et al, 1971); this has been taken by some to suggest that an additional environmental risk may be operating which could partly explain the familial associations that have been observed. Alternatively, genetic predisposition to chromosomal aberrations has been proposed to underlie familial clustering of BL (Wurster-Hill et al, 1974; Cervanka et al, 1977; Brubaker et al, 1980). The presence of a chromosome 8 to 14 translocation in BL tumour cells indicates a cytogenetic basis for cell transformation (Leder, 1976). These findings and the early childhood infection with Epstein–Barr virus (EBV) (de-The, 1979) have been linked in a model which describes the evolution of BL cells (Lenoir et al, 1987; Klein, 1979). No specific HLA associations have been identified with BL (Tiwari, 1985), although a link has been reported between class I MHC antigens and nasopharyngeal carcinoma, the other EBV-associated tumour (Lu et al, 1990).

In this report we describe the first evidence of familial BL outside Africa in Papua New Guinea.

*Received 3 November 1995*
*Revised 23 September 1996*
*Accepted 23 September 1996*

*Correspondence to:* BJ Brabin, Liverpool School of Tropical Medicine, Pembroke Place, Liverpool, L3 5QA, UK

### METHODS

The cases in this study were identified during the course of a large epidemiological study of BL in PNG from 1958 to 1987. Records were accessed from the National Tumour Registry in Port Moresby, the National Cancer Treatment Centre in Lae and from provincial hospitals and regional health centres in Madang and the East and West Sepik provinces. These provinces represent those three of the 18 provinces in PNG for which personal follow-up of case reports was possible. Families of BL cases in Madang and East and West Sepik provinces were interviewed to obtain accurate histories of family relationships. All cases were mapped for which the home location was precisely known. We assessed whether the rate of occurrence of sib groups might reflect the play of chance using the approach of Brubaker et al (1980). A chi-squared statistic for heterogeneity of the incidence between provinces and also between districts within a province was calculated. Seasonal analysis of monthly frequencies was calculated using Edward's harmonic analysis (Edwards, 1961).

The PNG Tumour Registry included cases with an established clinical and/or histological diagnosis. Clinical diagnostic criteria for BL are considered adequate in regions where the disease accounts for a large proportion of childhood cancers (Wright, 1967). In this study, a clinically defined case, for which histological confirmation was not available, had to fulfil the following criteria: a rapidly growing facial and/or abdominal tumour, no response to antibiotics (including tuberculosis therapy) and rapid initial response to chemotherapy (Ziegler et al, 1971).

### RESULTS

Among 174 cases of BL in West Sepik, East Sepik and Madang Provinces, three families were identified in which two or more cases of BL had occurred (Figure 1 and Table 2). Two families were identified from medical records and one was identified at interview with BL families. In family 1, three cases of BL occurred between 1964 and 1965 in full siblings. This was the only family with children affected in their locality. In family 2, BL occurred

**Table 1** Previous reports of familial BL and other neoplasms from Africa

| Reference | Area | Period | Features |
|---|---|---|---|
| Dalldorf et al (1964) | Kenya | Early 1960s | Two sibling with BL |
| Wright (1967) | East Africa | Early 1960s | Siblings with BL in two families |
| Morrow et al (1971) | Uganda | 1966–1968 | Siblings with BL in two families |
| Williams et al (1978) | Uganda | 1961–1975 | BL in siblings; BL in cousins in seven families |
| Brubaker et al (1980) | Tanzania | 1959–1972 | BL in three siblings in four families; two siblings one BL, one CML; mother NPC, child BL; grandfather NPC, child BL |

CML, chronic myeloid leukaemia; NPC, nasopharyngeal carcinoma.

**Figure 1** Pedigrees of families 1, 2 and 3. □, male; ○ female. Symbol filled in: BL cases. Symbol crossed through: dead within a year (1983–84) in siblings. In family 3, descended from one man and two wives, three cases of BL arose between 1980 and 1983. All family members lived within 2 km of each other and two of the cases in one family occurred within a period of 6 months. Other deaths in this family were not attributable to BL. Histological confirmation of diagnosis was available for four of these cases; for five, clinical diagnoses had been made in provincial hospitals, and the remaining diagnoses were made retrospectively by one of the authors (AW), using the criteria described above (Ziegler et al, 1971). Seven of these children received no treatment and died within one year of presentation. Of the two cases treated with chemotherapy, one had survived at least 1 year.

There was significant variation in the annual average incidence of BL among the three provinces studied ($P < 0.001$, Table 3). The rates in the Lumi and Nuku census districts of West Sepik province were remarkably high (9.03 and 18.70 per $10^5$) and far higher than those in the adjacent East Sepik Province. These differences remain when data from the Tumour Registry alone were used (Lumi 4.5 per $10^5$, Nuku 8.5 per $10^5$ and East Sepik 0.7 per $10^5$).

Table 4 gives the distribution of cases by month of onset. There was no evidence of a seasonal peak in frequency for the study population as a whole using Edward's harmonic analysis ($P$ = 0.47). In addition, there was no significant monthly variation in BL in the Lumi and Nuku districts (census district 5 and 6, Table 3)

© Cancer Research Campaign 1997

**Table 2** Details of PNG children with family histories of BL

| Family | Sex | Age (years) | Related | Site | Hospital | Treatment | Onset | Outcome |
|---|---|---|---|---|---|---|---|---|
| 1 | M | 3 | Brother | Facial | None | None | 1964 | D 1964 |
| | M | 2 | Brother | Facial | None | None | 1964 | D 1964 |
| | M | 12 | Index case[a] | Facial | Lumi, Sepik | None | 1965 | D 1965 |
| 2 | M | 10 | Brother[a] | Facial | Madang | Chemotherapy | 1983 | D 1984 |
| | F | 6 | Sister[a] | Liver | Madang | None | 1984 | D 1985 |
| 3 | M | 6 | Half brother | Facial | Kafle, Sepik | None | 1980 | D 1980 |
| | M | 3 | Index case[a] | Facial | Wewak, Sepik | Chemotherapy | 1982 | A 1987 |
| | F | 4 | Niece | Facial | None | None | 1983 | D 1983 |

[a]Cases identified from the Tumour Registry. D, died; A, alive.

**Table 3** Annual average incidence rates per 100 000 (1958–87) in subjects aged 0–17 years in West Sepik Province, East Sepik Province and Madang Province PNG

| Province Census district no. | Census district name | Total population (aged 0–17 years) | Cases aged (0–17 years) | Annual average rate per $10^5$ | 95% | CI |
|---|---|---|---|---|---|---|
| West Sepik Province | | 42 305 | 65 | 5.30 | 4.10 | 6.69 |
| 1 | Vanimo | 4 063 | 3 | 2.54 | 0.63 | 6.60 |
| 2 | Amanab | 8 559 | 11 | 4.43 | 2.30 | 7.59 |
| 3 | Telfomin | 8 007 | 2 | 0.86 | 0.14 | 2.66 |
| 4 | Aitape | 8 198 | 8 | 3.36 | 1.32 | 5.36 |
| 5 | Lumi | 11 450 | 30 | 9.03 | 6.18 | 12.66 |
| 6 | Nuku | 2 028 | 11 | 18.70 | 9.72 | 31.97 |
| East Sepik Province | | 92 308 | 33 | 1.23 | 0.86 | 1.70 |
| 7 | Maprik | 45 160 | 19 | 1.45 | 0.89 | 2.20 |
| 8 | Ambunti | 13 252 | 2 | 0.52 | 0.09 | 1.61 |
| 9 | Wewak | 16 710 | 8 | 1.65 | 0.75 | 3.07 |
| 10 | Angoram | 17 186 | 4 | 0.80 | 0.25 | 1.86 |
| Madang Province | | 89 368 | 76 | 2.93 | 2.32 | 3.64 |
| 11 | Bogia | 18 755 | 16 | 2.94 | 1.73 | 4.63 |
| 12 | Ramu | 25 441 | 12 | 1.63 | 0.87 | 2.73 |
| 13 | Madang | 33 415 | 37 | 3.82 | 2.75 | 5.18 |
| 14 | Rai | 11 757 | 11 | 3.23 | 1.68 | 5.52 |

Difference between all census districts, $P<0.001$. CI, confidence interval.

**Table 4** Diagnosis of Burkitt's lymphoma in West Sepik Province, East Sepik and Madang Provinces from 1958 to 1987 by month

| Month of diagnosis | BL cases |
|---|---|
| Jan | 18 |
| Feb | 14 |
| Mar | 11 |
| Apr | 6 |
| May | 18 |
| June | 15 |
| July | 15 |
| Aug | 13 |
| Sept | 14 |
| Oct | 20 |
| Nov | 12 |
| Dec | 10 |

Month of diagnosis not recorded in nine cases. Edward harmonic analysis: peak–low ratio = 1.31; harmonic peak (256 degrees), mid-August; $P = 0.472$.

where incidence was high and climate, communications and health services are likely to be similar.

Figure 2 shows the spatial distribution of cases from the three provinces. Many of the cases occurred below 300 m altitude. There was no significant variation in BL incidence by year of diagnosis. However, there were several case pairs documented, some occurring in the same family (see above). The only two cases reported in the 29 years of the study period from the island of Bagbag (census district 13) occurred within months of each other and were non-related (Figure 2).

Other families were identified for whom personal follow-up was not possible. One, involving a pair of siblings, was from Morobe Province, which is outside the study area, and was identified from the Tumour Registry. This sibling pair occurred within a year of each other (1983–84), but the pedigree of the family is unknown. One child was female (11 years) and one male (4 years). A further two families were found in which one sibling had developed BL while the other developed Hodgkin's disease; one of the sibling pairs was identified from the patients' medical records and the other from a note in the tumour registry.

### DISCUSSION

Heterogeneity in family risk may be related to variation in genetic susceptibility, phenotypic risk factors and environmental exposures (Schwartz, 1991). The present findings, in conjunction with earlier reports of familial BL in Africa, suggest that genetic factors play some role in the pathogenesis of this tumour. The relative infrequency of familial BL in PNG and Africa suggests that

**Figure 2** Map of West Sepik, East Sepik and Madang provinces showing the location of BL cases occurring from 1958 to 1987. ●, Histologically confirmed cases; ○, clinically diagnosed cases; x, 'uncertain' cases. Numbers refer to census district numbers shown in Table 3. West Sepik – numbers 1 to 6; East Sepik numbers 7 to 10; Madang numbers 11 to 14. Areas marked in darker tone are more than 300 m above sea level genetic susceptibility would be strongly modulated by environmental factors. Because of the established relationship between BL and holoendemic *P. falciparum* malaria, the latter must play an important modulatory role. The clinical severity of malaria has also been linked to host HLA specificities (Watier, 1993) and the familial occurrence of BL may be governed by this relationship between the major histocompatibility complex and *P. falciparum* malaria.

Even if there is genetic susceptibility, why should the time of onset of these cases cluster in children of widely differing ages? Space–time clustering of BL cases has been described in Uganda (Morrow et al, 1971; Williams et al, 1978). However, no evidence of clustering was found in another study in Uganda (Morrow et al, 1976) or in the north Mara District of Tanzania (Brubaker et al, 1973). A fall in incidence over time may lead to a failure to detect such clustering. For example, the disappearance of clustering effects from some areas could be explained by postulating factors that 'moved out' temporarily, coinciding with each other at the time of the clustering leaving an 'immune' population behind. Information from cluster studies has been used to calculate a latent period between the occurrence of a precipitating factor and onset of disease (Day et al, 1985). This latent period was found to be less than 1 year on average, and rarely to exceed 2 years.

In the present study, incident cases in families occurred within 1 year, which suggests an environmental factor affecting relatives at the same time. Spraying with DDT to reduce malaria transmission has been used in PNG since the late 1950s and reportedly had an effect on BL incidence (Peters and Standfast, 1960). The Madang and West Sepik, areas of high BL incidence, are, however, two provinces which have never been sprayed against malaria (Henderson and Aiken, 1979). As all children in these areas would have been repeatedly exposed to *P. falciparum* then perhaps another agent is involved, leading to EBV activation and the development of the tumour in a subgroup of children, and one which is acting at the micro level and is dependent on the exact locations at which the familial cases occurred. An association has been reported between the presence of certain plants possessing EBV-activators and the homes of BL patients suggesting that the former may influence incidence of BL at a micro level (Van den Bosch et al, 1993). However, in the absence of more specific hypotheses it seems unlikely that space–time cluster analysis will be of value (Wartenburg, 1995).

Thus, our data show that familial clustering of BL occurs in PNG as well as in Africa. These groupings were very close in time and space, as was the occurrence in one non-related pair. There was also spatial variation in incidence rates at provincial and district levels, but no large scale changes with time or season were identified.

### ACKNOWLEDGEMENTS

We are grateful to Mezza Ginny and Joe Paino of the Papua New Guinea Institute of Medical Research for their assistance with field visits and to the Director and staff of the National Tumour Registry, Port Moresby, and the staff of the National Cancer Treatment Centre, Lae, and of provincial hospitals and regional

© Cancer Research Campaign 1997

health centres in Madang and East and West Sepik provinces. Dr Thomas was supported by the National Health and Medical Research Council of Australia and the Queensland Institute of Medical Research.

### REFERENCES

Brubaker G, Geser A and Pike MC (1973) Burkitt's lymphoma in the north Mara District of Tanzania 1964–70: failure to find evidence of time-space clustering in a high risk isolated rural area. *Br J Cancer* **18**: 469–472

Brubaker G, Levin AG, Steel CM, Creasey G, Cameron HM, Linsell CA and Smith PG (1980) Multiple cases of Burkitt's lymphoma and other neoplasms in families in the North Mara district of Tanzania. *Int J Cancer* **26**: 165–170

Campbell PE and Reid IS (1974) Cancer in children in Papua New Guinea. In *The Epidemiology of Cancer in Papua New Guinea*, Atkinson GL, Clezy JK, Reay-Young PS, Scott GC and Wigley SC (eds), pp. 139–148 Department of Public Health: Konedobu.

Cervanka J, Anderson RS, Nesbit ME and Kirvit W (1977) Familial leukaemia and inherited chromosome aberrations. *Int J Cancer* **19**: 783–788

Dalldorf G, Linsel CA, Barnhart FE and Martyn R (1964) An epidemiological approach to the lymphomas of African children and Burkitt's sarcoma of the jaws. *Perspect Biol Med* **7**: 435–449

Day NE, Smith PG and Lachet B (1985) The latent period of Burkitt's lymphoma: the evidence from epidemiological clustering. In *Burkitt's Lymphoma: A human cancer model*, Lenoir GM, O'Conor GT and Olweny CLM (eds), pp. 187–195. International Agency for Research on Cancer Scientific Publications: Lyon de-The G (1979) The epidemiology of Burkitt's lymphoma: evidence for a causal association with Epstein–Barr virus. *Epidemiol Rev* **1**: 32–54

Edwards JH (1961) The recognition and estimation of cyclic trends. *Ann Hum Gen* **25**: 83–87

Henderson BE and Aiken GH (1979) Cancer in Papua New Guinea. *Nat Cancer Inst Monogr*, **53**: 67–72

Klein G (1979) Lymphoma development in mice and humans: Diversity of initiation is followed by convergent cytogenetic evolution. *Proc Natl Acad Science USA* **76**: 2442–2446

Leder P (1985) Translocations among antibody genes in human cancer. In *Burkitt's Lymphoma: A Human Cancer Model*, Lenoir GM, O'Conor GT and Olweny CLM (eds), pp. 341–358 Scientific Publications No. 60. International Agency for Research on Cancer: Lyon.

Lenoir GM and Bornkamm GW (1987) Burkitt's lymphoma, a human cancer model for the study of multistep development of cancer: proposal for a new scenario. *Adv Viral Oncol* **7**: 173–206

Lu S, Day NE, Degos L, Lepage V, Wang P, Chan SH, Simons M, McKnight B, Easton D, Zeng Y and deThe G (1990) Linkage of a nasopharyngeal carcinoma susceptibility locus to the HLA region. *Nature* **346**: 470–471

Morrow RH, Kisuule A, Pike MC and Smith PG (1976) Burkitt's lymphoma in the Mengo District of Uganda: epidemiologic features and their relationship to malaria. *J Natl Cancer Inst* **56**: 479–483

Morrow RH, Pike MC, Smith PG, Ziegler JL and Kisuule A (1971) Burkitt's lymphoma: a time-space cluster of cases in Bwamba County of Uganda. *B Med J* **2**: 491–492

Peters W and Standfast HA (1960) Studies on the epidemiology of malaria in New Guinea. *Transactions of the Royal Society of Tropical Medicine and Hygiene* **54**: 242–260

Schwartz AG, Kaufmann R and Moll PP (1991) Heterogeneity of breast cancer risk in families of young breast cancer patients and controls. *Am J Epidemiol* **134**: 1325–34

Tiwari JL and Terasaki PI (1985) *HLA and Disease Associations*. Springer-Verlag: New York, Berlin, Tokyo

Van den Bosch C, Griffin BE, Kazembe P, Dziweni C and Kadzamira L (1993) Are plant factors a missing link in the evolution of endemic Burkitt's lymphoma. *Br J Cancer* **68**: 1232–1235

Wartenberg D (1995) Should we boost or bust cluster investigations? *Epidemiology* **6**: 575–576

Watier H, Auriault C and Capron A (1993) Does Epstein–Barr virus infection confer selective advantage to malaria-infected children. *Lancet* **341**: 612–613

Williams EH, Smith PG, Day NE, Geser A, Ellice J and Tukei P (1978) Space-time clustering of Burkitt's lymphoma in the West Nile District of Uganda: 1961–1975. *Br J Cancer* **37**: 109–122

Wright DH (1967) The epidemiology of Burkitt's tumour. *Cancer Res* **27**: 2424–2438

Wurster-Hill DH, Cornwell GG and McIntyre OR (1974) Chromosomal aberrations and neoplasm – a family study. *Cancer* **33**: 72–81

Ziegler JL, Wright DH and Kyalwazi SK (1971) Differential diagnosis of Burkitt's lymphoma of the face and jaws. *Cancer* **27**: 503–514

© Cancer Research Campaign 1997